# Prematurity Modifies the Risk of Long-term Neurodevelopmental Impairments After Invasive Group B *Streptococcus* Infections During Infancy in Denmark and the Netherlands

**DOI:** 10.1093/cid/ciab774

**Published:** 2021-11-03

**Authors:** Erzsébet Horváth-Puhó, Linde Snoek, Merel N van Kassel, Bronner P Gonçalves, Jaya Chandna, Simon R Procter, Diederik van de Beek, Brechje de Gier, Arie van der Ende, Henrik T Sørensen, Joy E Lawn, Merijn W Bijlsma, GBS Danish and Dutch Collaborative Group for Long-term Outcomes, Henrik T Sørensen, Erzsébet Horváth-Puhó, Kirstine K Søgaard, Diederik van de Beek, Merijn W Bijlsma, Merel N van Kassel, Linde Snoek, Brechje de Gier, Arie van der Ende, Susan J M Hahné

**Affiliations:** 1 Department of Clinical Epidemiology, Aarhus University, Aarhus N, Denmark; 2 Department of Neurology, Amsterdam Neuroscience, Amsterdam University Medical Centers, University of Amsterdam, Amsterdam, The Netherlands; 3 Maternal, Adolescent, Reproductive and Child Health (MARCH) Centre, London School of Hygiene and Tropical Medicine, London, United Kingdom; 4 Department of Infectious Disease Epidemiology, London School of Hygiene and Tropical Medicine, London, United Kingdom; 5 Centre for Infectious Disease Control, National Institute for Public Health and the Environment, Bilthoven, The Netherlands; 6 Department of Medical Microbiology and Infection Prevention, Amsterdam University Medical Centers, University of Amsterdam, Amsterdam, The Netherlands; 7 Netherlands Reference Laboratory for Bacterial Meningitis, Amsterdam University Medical Centers, National Institute for Public Health and the Environment, Amsterdam, The Netherlands; 8 Department of Paediatrics, Amsterdam Infection and Immunity, Amsterdam University Medical Centers, University of Amsterdam, Amsterdam, The Netherlands

**Keywords:** *Streptococcus agalactiae*, group B *Streptococcus*, neurodevelopmental impairment, gestational age, effect modification

## Abstract

**Background:**

Preterm birth and neonatal infections are both associated with mortality and long-term neurodevelopmental impairments (NDIs). We examined whether the effect of invasive group B *Streptococcus* disease (iGBS) on mortality and long-term NDI differs for preterm and term infants, and whether co-occurrence of iGBS and prematurity leads to worse outcome.

**Methods:**

Nationwide cohort studies of children with a history of iGBS were conducted using Danish and Dutch medical databases. Comparison cohorts of children without iGBS were matched on birth year/month, sex, and gestational age. Effects of iGBS on all-cause mortality and NDI were analyzed using Cox proportional hazards and logistic regression. Effect modification by prematurity was evaluated on additive and multiplicative scales.

**Results:**

We identified 487 preterm and 1642 term children with a history of iGBS and 21 172 matched comparators. Dutch preterm children exposed to iGBS had the highest mortality rate by 3 months of age (671/1000 [95% CI, 412–929/1000] person-years). Approximately 30% of this mortality rate could be due to the common effect of iGBS and prematurity. Preterm children with iGBS had the highest NDI risk (8.8% in Denmark, 9.0% in the Netherlands). Of this NDI risk 36% (Denmark) and 60% (the Netherlands) might be due to the combined effect of iGBS and prematurity.

**Conclusions:**

Prematurity is associated with iGBS development. Our study shows that it also negatively impacts outcomes of children who survive iGBS. Preterm infants would benefit from additional approaches to prevent maternal GBS colonization, as this decreases risk of both preterm birth and iGBS.

**KEY FINDINGS**

**1. What Is Known and What Is New?**

Group B *Streptococcus* (GBS) carriage is a risk factor for premature birth as well as for invasive GBS disease (iGBS). Both prematurity and infections are leading causes of neonatal mortality and morbidity worldwide. An important knowledge gap exists regarding the combined influence of prematurity and iGBS. In particular, data are lacking on the risk of long-term neurodevelopmental impairments (NDIs) after iGBS in term and preterm children.

**2. What Did We Do and What Did We Find?**

National healthcare databases in Denmark (1997–2017) and the Netherlands (2000–2017) were used to generate a cohort of 487 preterm and 1642 term children with a history of iGBS and a comparison cohort of 21 172 children without iGBS (4752 preterm and 16 420 term children) to study mortality and long-term neurodevelopmental outcomes. Compared with term children without an iGBS history, higher risks of NDIs and need for educational support were observed in preterm children without iGBS, in term children with iGBS, and in preterm children with iGBS at the ages of 5 and 10 years. We found that prematurity influences the effect of iGBS history on NDI risk: 36% (Denmark) and 60% (the Netherlands) of the risk in preterm children with iGBS could be due to the combined effect of iGBS and prematurity.

**3. What To Do Now in ProgramMES?**

Preterm infants are at increased risk of early mortality and long-term NDIs. These risks are amplified after contracting iGBS. This is particularly worrying as prematurity has been associated with increased risk of developing iGBS itself, and GBS colonization is a risk factor for preterm birth. Approaches to preventing, treating, and improving follow-up of infants with iGBS could be particularly beneficial for preterm infants.

**4. What Next for Research?**

Prematurity and iGBS are global public health problems. In low- and middle-income countries, the burden of prematurity and iGBS is higher than in affluent countries and they may co-occur more often. Data from resource-limited settings are needed to better understand how these conditions impact each other’s effect on children’s health. Recent estimates of the global burden of iGBS highlight the importance of quantifying long-term sequelae, since they have a significant impact on affected children and families. Therefore, addressing this data gap is all the more urgent.

Advances in perinatal care have led to improved survival of premature infants. Prematurity is a major public health problem in all countries, occurring in 5–18% of all births [1, 2]. Improving long-term outcome of children born preterm has become a global priority [3]. Preterm infants are at higher risk of neonatal death [4, 5] and long-term neurological outcome disorders spanning the motor, sensorial, cognitive, and behavioral domains [6, 7]. While group B *Streptococcus* (GBS; *Streptococcus agalactiae*) colonization increases the risk of preterm birth [8], prematurity is also a known risk factor for bacterial infections during infancy, including both early- and late-onset GBS disease [8–10]. Invasive GBS disease (iGBS) in itself is associated with a considerable risk of mortality [11, 12] and long-term sequelae: iGBS survivors experience higher risk of long-term neurodevelopmental impairments (NDIs). Based on a meta-analysis [13], on average, 18% (95% confidence interval [CI], 13–22%) of children have been diagnosed with moderate or severe NDIs after GBS meningitis. A recent cohort study found that 3.9% of children were diagnosed with moderate or severe NDI after GBS sepsis [14]. Besides the fact that infants with iGBS will more often be preterm, the co-occurrence of these 2 conditions might lead to particularly poor outcomes. However, despite these potentially severe consequences for the lives of preterm children with iGBS, the extent to which prematurity modifies iGBS-linked outcomes remains to be quantified.

This paper is part of a series Every Country, Every Family: Group B Streptococcal Disease Worldwide. Our current objectives are as follows: (1) describe the clinical characteristics of term and preterm children with iGBS infection, (2) examine the effect of iGBS on mortality by prematurity status and the effect of iGBS and prematurity on death using a common reference category, and (3) estimate the effect of iGBS on NDIs by prematurity and the effect of iGBS and prematurity on long-term NDI outcomes using a common reference category. Our overarching goal is to improve clinical care by identifying clinically relevant factors that explain variation in the health outcomes of children with iGBS. As part of this effort, another paper in this series focuses on the influence of sex on the long-term outcomes after iGBS (Kassel et al) [15].

## METHODS

### Study Design

**Figure 1.**
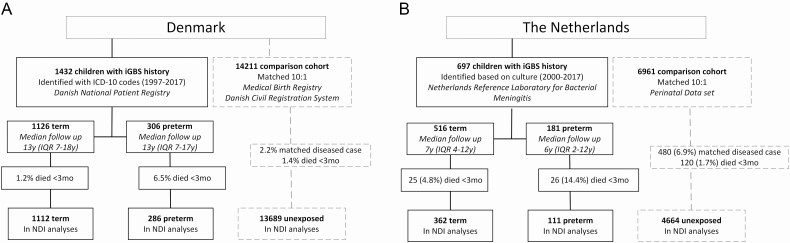
Flowchart of study population selection in Denmark (*A*) and the Netherlands (*B*). Abbreviations: iGBS, invasive group B *Streptococcus* disease; ICD-10, *International Classification of Diseases, 10th revision*; IQR, interquartile range; NDI, neurodevelopmental impairment.

We used Danish population-based medical and administrative registries (covering the years 1997–2017) and Dutch population and bacterial surveillance registries (covering the years 2000–2017) to conduct nationwide matched cohort studies, as previously described [14]. Children with iGBS were defined as having a history of iGBS (GBS sepsis or meningitis) by the age of 89 days (Figure 1). In Denmark, which has a free, tax-supported healthcare system [16, 17], these children were identified based on *International Classification of Diseases, 10th revision* (ICD-10), codes of discharge diagnoses from the Danish National Patient Registry (Supplementary Table 1) [18–20]. This registry contains data on dates of admission and discharge from all Danish hospitals, emergency room visits, and outpatient clinic visits since 1995. In the Netherlands, children with iGBS were defined as those with cerebrospinal fluid and/or a blood culture positive for GBS, identified through the Netherlands Reference Laboratory for Bacterial Meningitis. This laboratory receives from microbiology laboratories approximately 90% of isolates cultured from the blood or cerebrospinal fluid of infants with invasive infection [21, 22]. Children without a history of iGBS were randomly selected to form a comparison cohort matched 10:1 with cases on sex, birth year/month, and gestational age (<28 weeks, 28–36 weeks, and ≥37 weeks) using the Danish Medical Birth Registry and the Danish Civil Registration System in Denmark and the PeriNed perinatal registry and the Municipal Personal Records Database in the Netherlands [23, 24].

### Outcome Data

Our first objective was to examine clinical characteristics of term and preterm children with iGBS. For each child, information on sex, year of birth, iGBS clinical syndrome (meningitis, sepsis), and gestational age was obtained from the population registries described above. In the Netherlands, iGBS was categorized as either early onset (at the age of 0–6 days) or late onset (at the age of 7–89 days). In Denmark, the timing of disease onset could not be reliably determined by the date of admission for children with late-onset iGBS because they could have already been admitted to the hospital for other reasons before developing the infection. The second objective was to study the effect of iGBS on mortality by prematurity and to analyze the combined effect of iGBS and prematurity on death. The Danish Civil Registration System [19] and the Dutch Municipal Personal Records Database [23] were used to assess all-cause mortality during the first 89 days and the first 5 years of life. The third objective was to examine the effect of iGBS on NDIs by prematurity and to analyze the effect of iGBS and prematurity on long-term NDI outcomes using a common reference category. In Denmark, diagnoses of NDI were based on ICD-10 codes, obtained from the Danish National Patient Registry [20]. Motor, hearing, vision, cognitive, and social/behavioral domains were studied and impairments were categorized by severity (mild/moderate/severe). This registry records information mainly on severe cases, omitting children with milder forms of NDI monitored only by the school system or by general practitioners. In the Netherlands, we used data on special educational support recorded in national school registries as a surrogate marker for NDI. Children who received education in special-needs schools were classified as having “moderate/severe NDI” and those who received additional support in regular schools were classified as having “mild NDI.” Detailed definitions of outcome variables have been provided in a previous publication [14].

### Statistical Analyses

Results of descriptive analyses and outcome data are presented for each country separately. All children were followed from birth until death, emigration, or until the end of the study (31 December 2017), whichever occurred first. We assessed overall mortality by calculating mortality risk during the first 3 months and the first 5 years of life and mortality rates per 1000 person-years. Hazard ratios (HRs) and 95% confidence intervals (CIs) were estimated using Cox proportional hazards regression after adjusting for sex and birth year. We evaluated the extent to which gestational age modified the effect of iGBS on overall mortality on both additive and multiplicative scales (Objective 2, Supplementary Table 2) [25, 26]. Effect modification on the additive scale was examined using mortality rates, first by calculating the interaction contrast and then by estimating HRs based on the common reference group of non-iGBS term children (analyses with a common reference group). An interaction contrast is a measure of the departure of mortality rates from an additive model. It is calculated as the difference between rate differences in strata with and without prematurity, as follows: interaction contrast = (mortality rate_iGBS, preterm_ – mortality rate_non-iGBS, preterm_) – (mortality rate_iGBS, term_ – mortality rate_non-iGBS, term_). In addition, we calculated attributable proportions (= interaction contrast/mortality rate in preterm children with iGBS), which measure the proportion of the mortality risk in preterm children with iGBS due to the combined effect of iGBS and prematurity. On the multiplicative scale, a modification occurs if relative association measures (ie, HRs) between exposure and outcome vary by strata of a third variable. We therefore performed stratified analyses by gestational age and assessed the effect modification on the multiplicative scale by including the product term (gestational age × iGBS) in multivariable Cox regression models.

Risks of NDI and need for special education were assessed at the ages of 5 and 10 years. The analyses included only those children followed until at least the corresponding cutoff age. The association between iGBS and NDI was assessed using logistic regression models; estimated odds ratios (ORs) were adjusted for year of birth and sex. We assessed whether gestational age modified the effect of iGBS on NDI outcomes on both additive and multiplicative scales (objective 3). Effect modification on the additive scale was examined by calculating the relative excess risk due to interaction (RERI), using the common reference group of term non-iGBS children (RERI = OR_iGBS, preterm_ – OR_iGBS__, term_ – OR_non-iGBS, preterm_ + 1). In addition, we calculated attributable proportions (= RERI/OR_iGBS, preterm_). We analyzed the association between iGBS and NDI outcomes stratified by gestational age and assessed the effect modification on the multiplicative scale by including the product term “gestational age × iGBS” in the logistic regression models.

Analyses were conducted using SAS version 9.4 (Denmark; SAS Institute) and SPSS Statistics version 25.0 (IBM Corporation) and STATA version 16 (The Netherlands; StataCorp).

## RESULTS

### Objective **1: Clinical Characteristics of iGBS and Non-iGBS Children**

We identified 2129 children with a history of iGBS, of whom 487 (22.9%) were born preterm (ie, before 37 weeks of gestation) and 1642 (77.1%) were born term (Table 1). Late-onset iGBS was more common in preterm children (95/181 [52.5%]) compared with term children (157/516 [30.4%]) in the Netherlands.

**Table 1.** Characteristics of Preterm and Term Children With iGBS and Members of Matched Comparison Cohorts in Denmark and the Netherlands

	Denmark				The Netherlands			
	Preterm		Term		Preterm		Term	
	non-iGBS	iGBS	non-iGBS	iGBS	non-iGBS	iGBS	non-iGBS	iGBS
Total	2951 (100)	306 (100)	11 260 (100)	1126 (100)	1801 (100)	181 (100)	5160 (100)	516 (100)
Period								
1997–1999	573 (19.4)	60 (19.6)	2430 (21.6)	243 (21.6)	…	…	…	…
2000–2005	1026 (34.8)	105 (34.3)	3570 (31.7)	357 (31.7)	454 (25.2)	46 (25.4)	1170 (22.7)	117 (22.7)
2006–2011	861 (29.2)	89 (29.1)	2760 (24.5)	276 (24.5)	477 (26.5)	48 (26.5)	1620 (31.4)	162 (31.4)
2012–2017	491 (16.6)	52 (17.0)	2500 (22.2)	250 (22.2)	870 (48.3)	87 (48.1)	2370 (45.9)	237 (45.9)
Sex								
Male	1609 (54.5)	166 (54.2)	6260 (55.6)	626 (55.6)	950 (52.7)	95 (52.5)	2930 (56.8)	293 (56.8)
Female	1342 (45.5)	140 (45.8)	5000 (44.4)	500 (44.4)	851 (47.3)	86 (47.5)	2230 (43.2)	223 (43.2)
Syndrome								
Meningitis	…	46 (15.0)	…	122 (10.8)	…	46 (25.4)	…	152 (29.5)
Sepsis	…	260 (85.0)	…	1004 (89.2)	…	135 (74.6)	…	364 (70.5)
Onset^a^								
EOD (< week)	…	…	…	…	…	86 (47.5)	…	359 (69.6)
LOD (≥1 week)	…	…	…	…	…	95 (52.5)	…	157 (30.4)
Gestational age								
<28 weeks	391 (13.2)	50 (16.3)	…	…	291 (16.2)	30 (16.6)	…	…
28–36 weeks	2560 (86.8)	256 (83.7)	…	…	1510 (83.8)	151 (83.4)	…	…
37+ weeks	…	…	11 260 (100)	1126 (100)	…	…	5160 (100)	516 (100)

Data are presented as n (%). Abbreviations: EOD, early-onset disease; iGBS, invasive group B *Streptococcus* disease; LOD, late-onset disease.

^a^In the Netherlands, the first reported date of illness (in most cases the first date a culture was taken [97.4%]) was used to calculate age of onset.

### Objective 2: Mortality

In Denmark, 1.2% (95% CI, .7–2.1%) of term and 6.5% (95% CI, 4.3–9.9%) of preterm children with iGBS died within 3 months after birth, compared to .1% (95% CI, .1–.2%) of term and 7.5% (95% CI, 6.6–8.5%) of preterm children without iGBS (Table 2). In the Netherlands, mortality risks in the iGBS cohort were higher, as 5.0% (95% CI, 3.3–7.1%) of term and 15.5% (95% CI, 10.0–20.4%) of preterm children with iGBS died within 3 months after birth, compared to .2% (95% CI, .1–.3%) and 6.4% (95% CI, 5.1–7.4%) of children without iGBS, respectively.

**Table 2.** Effect Modification of Prematurity on the Association Between iGBS and Mortality

		Analyses With a Common Reference Group		Stratified Analyses	
	Mortality Rate per 1000 PYs	HR^a^ (95% CI) for iGBS Using a Common Reference Group	Effect Modification on an Additive Scale,^b^ Interaction Contrast (95% CI); AP, %	HR^a^ (95% CI) for iGBS Within Strata of Gestational Age	Effect Modification on a Multiplicative Scale,^b^ *P* Value of iGBS × Prematurity Term
Denmark					
0–89 Days					
non-iGBS/term	4.0 (1.6–6.4)	1.00 (reference)	…	1.00 (reference)	…
iGBS/term	51.6 (24.6–78.6)	12.79 (5.81–28.17)	…	12.81 (5.81–28.21)	…
non-iGBS/preterm	330.6 (287.0–374.2)	79.10 (43.16–144.97)	…	1.00 (reference)	…
iGBS/preterm	283.8 (159.4–408.1)	67.88 (32.52–141.71)	−94.5 (−229.0, 40.1); N.A.	.86 (.54–1.36)	<.0001
0–5 Years					
non-iGBS/term	0.6 (0.4–0.8)	1.00 (reference)	…	1.00 (reference)	…
iGBS/term	3.1 (1.6–4.6)	5.19 (2.84–9.49)	…	5.22 (2.85–9.54)	…
non-iGBS/preterm	18.2 (15.9–20.6)	30.15 (20.73–43.87)	…	1.00 (reference)	…
iGBS/preterm	16.3 (9.5–23.1)	26.67 (15.44–46.07)	−4.4 (−11.8, 2.9); N.A.	.88 (.57–1.37)	<.0001
The Netherlands					
0–89 Days					
non-iGBS/term	7.2 (2.5–11.9)	1.00 (reference)	…	1.00 (reference)	…
iGBS/term	206.7 (125.7–287.8)	28.10 (13.12–60.20)	…	28.32 (13.22–60.67)	…
non-iGBS/preterm	268.3 (218.4–318.3)	36.51 (18.51–72.02)	…	1.00 (reference)	…
iGBS/preterm	671.3 (412.3–929.4)	86.71 (40.63–185.06)	203.4 (−71.7, 478.5); 30%	2.37 (1.54–3.63)	<.0001
0–5 Years					
non-iGBS/term	0.6 (0.3–1.0)	1.00 (reference)	…	1.00 (reference)	…
iGBS/term	12.5 (7.7–17.3)	18.08 (9.44–34.62)	…	18.16 (9.48–34.78)	…
non-iGBS/preterm	16.9 (13.8–19.9)	24.26 (13.94–42.23)	…	1.00 (reference)	…
iGBS/preterm	41.9 (26.1–57.7)	53.13 (27.85–101.37)	13.2 (−3.6, 30.0); 32%	2.18 (1.44–3.32)	<.0001

Abbreviations: AP, attributable proportion; CI, confidence interval; HR, hazard ratio; iGBS, invasive group B *Streptococcus* disease; N.A., not applicable; PY, person-year.

^a^HRs were adjusted for birth year and sex.

^b^Effect modification by gestational age on the additive scale tells us, for example, whether preventative measures are more effective in reducing mortality if applied to preterm or term children. While this especially addresses public health–related questions, effect modification on the multiplicative scale is more relevant to pediatricians and other clinicians because it quantifies whether relative associations between iGBS and mortality depend on the child’s gestational age. There may be a positive interaction on the additive scale but a negative or null interaction on a multiplicative scale, ie, the effect of both prematurity and iGBS on the rate difference scale may exceed the sum of the effects on the rate difference scale of prematurity and iGBS each considered separately, while the HR for prematurity and iGBS together may be less than the product of the effects of the 2 factors considered separately.

In the analysis of mortality among term children, we found that children with an iGBS history were more likely to die than their non-iGBS–matched comparators (HR, 5.2 [95% CI, 2.9–9.5] by 5 years of age in Denmark; HR, 18.2 [95% CI, 9.5–34.8] by 5 years of age in the Netherlands) (Table 2). In the Netherlands, preterm children with a history of iGBS were more likely to die compared to preterm children without iGBS (HR, 2.2; 95% CI, 1.4–3.3). In Denmark, this association was not observed (HR, .9; 95% CI, .6–1.4). The magnitude of the association was substantially reduced in both countries (Table 2). As suggested from the results described above (stratified analyses), there was evidence for effect modification by prematurity on a multiplicative scale in both countries (*P* values <.0001 at ages of 3 months and 5 years).

Compared with a common reference category of non-iGBS term children, all other categories were associated with higher mortality rates in the first 5 years of life (Table 2). In the Netherlands there was evidence for effect modification on the additive scale: Dutch preterm children with iGBS had the highest mortality rate in the first 3 months of life (671/1000 person-years; 95% CI, 412–929 person-years), and 30% of this mortality rate could be due to the combined effect of iGBS and prematurity (interaction contrast: 203; 95% CI, −72, 479). Due to the limited number of outcomes in Denmark, effect modification estimates on the additive scale were inconclusive.

Similar patterns were observed among children with a history of GBS sepsis and GBS meningitis and their comparison cohorts (Supplementary Table 3).

### Objective **3: Neurodevelopmental Impairments and Need for Special Educational Support**

**Figure 2.**
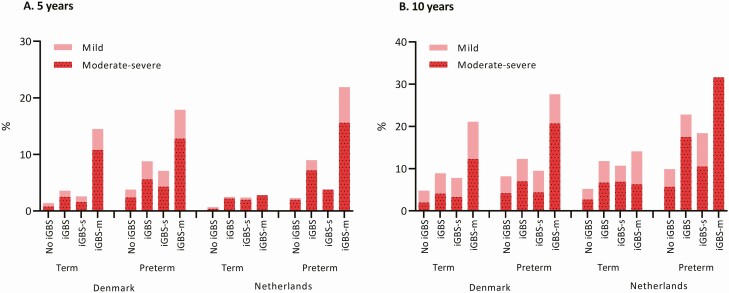
Proportion of children with neurodevelopmental impairments in Denmark and the Netherlands among 5-year survivors (*A*) and 10-year survivors (*B*). Abbreviations: iGBS, invasive group B *Streptococcus*; iGBS-m, iGBS meningitis; iGBS-s, iGBS sepsis.

In Denmark, 3.6% of term children with iGBS and 8.8% of preterm children with iGBS were diagnosed with NDIs of any severity by the age of 5 years, compared to 1.4% of term children without iGBS (OR, 2.7; 95% CI, 1.8–3.9) and to 3.8% of preterm children without iGBS (OR, 2.5; 95% CI, 1.5–4.0) (Figure 2, Table 3). In the Netherlands, 2.5% of term children with iGBS and 9.0% of preterm children with iGBS had NDI by the age of 5 years, compared to 0.8% of term children without iGBS (OR, 3.3; 95% CI, 1.5–7.0) and to 2.3% of preterm children without iGBS (OR, 4.2; 95% CI, 1.9–9.0) (Figure 2, Table 3). In both countries, there was no evidence of effect modification by prematurity for NDIs on the multiplicative scale.

**Table 3.** Effect Modification of Prematurity on Neurodevelopmental Impairment Outcomes after iGBS

		Analyses With a Common Reference Group		Stratified Analyses	
	NDI Proportion,% (95% CI)	OR^a^ (95% CI) for iGBS Using a Common Reference Group	Effect Modification on an Additive Scale,^b^ RERI (95% CI); AP, %	OR^a^ (95% CI) for iGBS Within Strata of Gestational Age	Effect Modification on a Multiplicative Scale,^b^*P* Value of iGBS × Prematurity Term
Denmark					
5 Years of age					
Any					
non-iGBS/term	1.4 (1.2–1.7)	1.00 (reference)	…	1.00 (reference)	…
iGBS/term	3.6 (2.5–5.1)	2.65 (1.81–3.89)	…	2.65 (1.81–3.89)	…
non-iGBS/preterm	3.8 (3.0–4.7)	2.78 (2.11–3.67)	…	1.00 (reference)	…
iGBS/preterm	8.8 (5.6–13.0)	6.89 (4.30–11.04)	2.46 (−.77, 5.68); 36%	2.47 (1.52–4.04)	.83
Mod-sev					
non-iGBS/term	.8 (.6–1.0)	1.00 (reference)	…	1.00 (reference)	…
iGBS/term	2.5 (1.6–3.7)	3.34 (2.08–5.38)	…	3.35 (2.08–5.39)	…
non-iGBS/preterm	2.4 (1.8–3.1)	3.23 (2.26–4.62)	…	1.00 (reference)	…
iGBS/preterm	5.6 (3.1–9.2)	7.95 (4.41–14.34)	2.38 (−2.24, 6.99); 30%	2.45 (1.34–4.48)	.44
10 Years of age					
Any					
non-iGBS/term	4.8 (4.3–5.3)	1.00 (reference)	…	1.00 (reference)	…
iGBS/term	8.9 (6.9–11.3)	1.97 (1.48–2.62)	…	1.97 (1.48–2.62)	…
non-iGBS/preterm	8.2 (6.9–9.6)	1.79 (1.46–2.20)	…	1.00 (reference)	…
iGBS/preterm	12.3 (8.0–17.9)	2.84 (1.80–4.46)	.07 (−1.32, 1.47); 2.5%	1.58 (.99–2.54)	.44
Mod-sev					
non-iGBS/term	2.0 (1.7–2.4)	1.00 (reference)	…	1.00 (reference)	…
iGBS/term	4.1 (2.7–5.9)	2.08 (1.37–3.15)	…	2.08 (1.37–3.15)	…
non-iGBS/preterm	4.2 (3.3–5.2)	2.10 (1.57–2.80)	…	1.00 (reference)	…
iGBS/preterm	7.0 (3.8–11.6)	3.60 (1.99–6.50)	.43 (−1.82, 2.67); 12%	1.72 (.93–3.17)	.62
The Netherlands					
5 Years of age					
Any					
non-iGBS/term	.8 (.5–1.1)	1.00 (reference)	…	1.00 (reference)	…
iGBS/term	2.5 (1.1–4.7)	3.26 (1.52–6.98)	…	3.27 (1.53–7.00)	…
non-iGBS/preterm	2.3 (1.5–3.5)	3.35 (1.93–5.83)	…	1.00 (reference)	…
iGBS/preterm	9.0 (4.4–15.9)	14.10 (6.60–30.14)	8.49 (−1.57, 18.55); 60%	4.17 (1.92–9.04)	P = .65
Mod-sev					
non-iGBS/term	.4 (.3–.7)	1.00 (reference)	…	1.00 (reference)	…
iGBS/term	2.2 (1.0–4.3)	5.08 (2.16–11.97)	…	5.10 (2.16–12.02)	…
non-iGBS/preterm	2.0 (1.3–3.1)	5.00 (2.59–9.64)	…	1.00 (reference)	…
iGBS/preterm	7.2 (3.2–13.7)	18.68 (7.77–44.93)	9.61 (−5.20, 24.41); 51%	3.71 (1.60–8.64)	.62
10 Years of age					
Any					
non-iGBS/term	5.2 (4.3–6.3)	1.00 (reference)	…	1.00 (reference)	…
iGBS/term	11.8 (7.6–17.2)	2.48 (1.53–4.03)	…	2.49 (1.53–4.06)	…
non-iGBS/preterm	10.0 (7.6–12.8)	2.21 (1.55–3.13)	…	1.00 (reference)	…
iGBS/preterm	22.8 (15.5–39.7)	6.28 (3.21–12.31)	2.60 (−1.64, 6.83); 41%	2.78 (1.39–5.56)	.75
Mod-sev					
non-iGBS/term	2.7 (2.1–3.5)	1.00 (reference)	…	1.00 (reference)	…
iGBS/term	6.7 (3.6–1.1)	2.58 (1.37-4.85)	…	2.60 (1.38–4.89)	…
non-iGBS/preterm	5.7 (3.9–8.0)	2.39 (1.51-3.79)	…	1.00 (reference)	…
iGBS/preterm	17.5 (8.8–29.9)	9.07 (4.23–19.44)	5.10 (−1.67, 11.86); 56%	3.68 (1.68–8.09)	.46

Abbreviations: AP, attributable proportion; CI, confidence interval; iGBS, invasive group B *Streptococcus* disease; mod-sev, moderate–severe; NDI, neurodevelopmental impairment; OR, odds ratio; RERI, relative risk due to interaction.

^a^ORs were adjusted for birth year and sex.

^b^Effect modification by gestational age on the additive scale indicates whether preventative measures are more effective in reducing NDI risk if applied to preterm or term children. While this especially addresses public health–related questions, effect modification on the multiplicative scale is more relevant to pediatricians and other clinicians because it quantifies whether relative associations between iGBS and NDI risk depend on the child’s gestational age. There may be a positive interaction on the additive scale but a negative or null interaction on a multiplicative scale, ie, the effect of both prematurity and iGBS on the risk difference scale may exceed the sum of the effects on the risk difference scale of prematurity and iGBS each considered separately, while the OR for prematurity and iGBS together may be less than the product of the effects of the 2 factors considered separately.

In both countries, a higher risk for NDIs (Denmark) and special educational support (The Netherlands) was observed in non-iGBS preterm, iGBS term, and iGBS preterm children compared with non-iGBS term children, both at the ages of 5 and 10 years (analyses with a common reference group) (Table 3). The effect modification on the additive scale was present at the age of 5 in both countries (RERI, 2.5 [95% CI, −.8, 5.7] in Denmark and RERI 8.5 [95% CI, −1.6, 18.6] in the Netherlands). At this age, 36% (in Denmark) and 60% (in the Netherlands) of the NDI risk in preterm children with iGBS could be due to the combined effect of iGBS and prematurity (Table 3, Supplementary Figure 1). Our results also provide evidence for effect modification on the additive scale for moderate and severe NDIs at age 5 (RERI of 2.4 in Denmark and RERI of 9.6 in the Netherlands). Similar patterns were observed among children with history of GBS sepsis and GBS meningitis (Supplementary Table 4). In Denmark, domain-specific data were suggestive of effect modification on the additive scale in social and motor domain–related NDIs (Supplementary Table 5).

## Discussion

It is well established that both prematurity and iGBS are independently associated with an increased risk of neonatal mortality and NDIs [7, 14, 27]. To our knowledge, this is the first study to assess whether the effect of iGBS on mortality and NDI outcomes is different for preterm compared with term infants (stratified analyses) and whether the combined effect of iGBS and prematurity leads to a worse outcome than would be expected based on the individual effects of iGBS and prematurity.

In the Netherlands, the effect of being born preterm and having iGBS led to a much higher mortality rate than prematurity or iGBS alone. Several biologically plausible factors might contribute to this pattern, including immaturity of the innate immune system [28] and a less efficient cardiovascular response in preterm children, resulting in more frequent hemodynamic instability during a period of infection [29–31]. Another contributing factor is the possible association between early-onset sepsis and the risk of intraventricular hemorrhage (IVH) [32–34], with mortality rates up to 40% [35]. Our data provide evidence for effect modification of mortality after iGBS by gestational age, on the additive scale in the Netherlands and on the multiplicative scale in both countries. The largest relative increase in mortality due to iGBS occurred in term children. This is likely due to the higher baseline mortality risk in preterm children, since preterm birth is the main global cause of neonatal death [36].

A notable difference between the 2 countries was seen in the higher mortality rates of children with iGBS in the Netherlands than in Denmark. The difference in case definitions might have contributed to this: since the identification of iGBS in Denmark was based on ICD-10 codes, it is likely that probable iGBS cases (ie, without positive culture) were included and it is known that mortality in this group is lower than culture-positive GBS cases [37]. We also note that a higher proportion of children with GBS meningitis was included in the Netherlands and mortality is highest in this group. A second difference is that, in contrast to the Netherlands, mortality rates in Denmark were highest in preterm children without iGBS. This can also be due to differences in the study cohorts. In Denmark, children with iGBS who died on the day of birth might have been missed.

In both countries, the absolute risk of NDIs was highest in children with a history of both prematurity and iGBS: for example, in the Dutch cohort by the age of 5 years, out of 100 preterm children with iGBS history, approximately 9 will be diagnosed with NDI (special education needs), with 5 of these being linked to effect modification by prematurity. In particular, the absolute risk of NDIs after GBS sepsis, the most common presentation of iGBS [11], was highest in preterm children. Preterm children are at increased risk of IVH, while associations between infection and IVH have also been reported. A recent meta-analysis showed an increased risk of moderate to severe NDIs among survivors of mild IVH (adjusted OR, 1.32; 95% CI, 1.09–1.77) [38]. In both countries, preterm children with GBS meningitis had the highest risk of moderate or severe NDI. This corresponds to the results of the meta-analysis on NDIs after GBS disease conducted by Kohli-Lynch et al [13]. However, this meta-analysis did not report results by prematurity and most of the studies included had fewer than 2 years of follow-up time. Our data provide evidence for effect modification of the risk of NDIs after iGBS by prematurity, on the additive scale in both countries.

The strengths of our study to address this important epidemiological question include its large sample size, its large number of comparator children without a history of iGBS, a multinational design, and long-term follow-up until adolescence. In particular, since assessment of effect modification involves estimation of effects in different strata (eg, in term and preterm children) a large cohort size is necessary. Another strength of our design is the different NDI definitions used in each study country. While the Danish results represent patients diagnosed with NDI in a hospital setting, the use of need for special educational support in the Netherlands can be interpreted as both a proxy for NDI, as well as a relevant functional outcome in its own right. Use of need for special education as a surrogate for NDI is supported by many studies that show that some early-life medical conditions increase the risk for both NDIs and need for special education [39–41].

The limitations of our study involve the differences in exposure (ie, GBS disease) ascertainment and missing information. Indeed, 1 concern is the difference in diagnosis of iGBS in the 2 countries: while ICD-10 codes were used in Denmark, only culture-positive infections were included in the Netherlands. In addition, children with iGBS who died on the day of birth might have been missed in both countries. Another limitation is that the number of very preterm children (gestational age <28 weeks) was small (50 children with iGBS in Denmark and 30 in the Netherlands). Data on gestational age in Denmark were retrieved from the Danish Medical Birth Registry, where missing data on gestational age at delivery decreased from 8% in 1997 to 1% in 2001 and has remained constant at that level since then. In the Netherlands, data on gestational age from the PeriNed perinatal registry were available only for children born during 2000–2017. Therefore, 5% of children with iGBS had to be excluded. Last, we did not control for other possible confounders, such as socioeconomic factors.

Group B *Streptococcus* colonization is associated with preterm birth [8], and preterm children are also at increased risk of GBS infection [9, 10]. Therefore, preventing GBS colonization has a double rationale: it decreases the risk of preterm birth and the risk of iGBS in newborns, including preterm infants, at the same time. The only prevention strategy currently available for iGBS is intrapartum antibiotic prophylaxis (IAP). However, this has no effect on GBS colonization and therefore does not prevent preterm births. Also, while prematurity is a major risk factor for late-onset iGBS, IAP does not prevent and might even increase the risk of late-onset infection [42, 43]. An intervention to prevent maternal GBS colonization during pregnancy has the potential to prevent preterm birth as well as iGBS. Methods for preventing GBS colonization in pregnant women are being developed. Among these are the use of oral probiotics and bacteriophages to reduce GBS colonization in pregnant women [44–46]. Another promising future strategy to prevent neonatal GBS infections is maternal GBS vaccination [47]. Besides preventing early- and late-onset iGBS, a multivalent maternal GBS vaccine also has the potential to reduce neonatal exposure to GBS by delaying new acquisition of maternal colonization [48]. However, transfer of protective maternal antibodies is more limited in preterm children [49] and this should be considered in studies assessing vaccine efficacy.

### Conclusions

Preterm children are at increased risk of iGBS and adverse outcomes. While NDI risk after iGBS has been previously described [14], the effect of prematurity on long-term risk of NDIs in children with iGBS had not been studied. Knowledge about factors that explain variation in health outcomes of children with iGBS is important for clinicians and educators, allowing them to improve and personalize clinical care and thereby minimize the impact of sequelae. The results of our study underscore the distressingly high risk of adverse outcomes in preterm children with iGBS and emphasize the need for preventive strategies countering GBS colonization, thereby decreasing the risk of preterm birth and iGBS. Our results also provide insights from a public health perspective. Global estimates of the GBS burden suggest that long-term sequelae linked to iGBS are an important public health problem [12], and our findings indicate that this burden is not homogenously distributed, even in a single setting; preterm infants are likely to represent an important proportion of worldwide NDI cases related to GBS. There is a need to collect and collate gestational age–stratified data on long-term outcomes after iGBS, especially including low- and middle-income countries. In that way, we could better inform global burden estimation and cost-effectiveness analyses of new interventions, such as maternal GBS vaccines.

## Supplementary Data

Supplementary materials are available at *Clinical Infectious Diseases* online. Consisting of data provided by the authors to benefit the reader, the posted materials are not copyedited and are the sole responsibility of the authors, so questions or comments should be addressed to the corresponding author.

ciab774_suppl_Supplementary_Materials

## Notes

***Authors’ contributions.*** J. E. L. conceived the idea for this study and J. E. L., E. H.-P., B. P. G., L. S., M. W. B., H. T. S., and M. N. v. K. designed the study protocol. Analyses were undertaken by E.H.-P. (Danish analyses) and M. N. v. K. (Dutch analyses). The manuscript was drafted by E. H.-P. and L. S. All authors reviewed and helped to revise the manuscript. All authors reviewed and agreed on the final version.

***Acknowledgments.*** We thank PeriNed and Statistics Netherlands for their cooperation and for making their valuable data available for this study.

***Ethics and consent to participate.*** The study was reported to the Danish Data Protection Agency (record no. 2015-57-0002). In the Netherlands, the study protocol (EPI-408) was submitted to the Centre for Clinical Expertise at the National Institute for Public Health and the Environment. It was exempted from further approval by an ethical research committee, according to Dutch law for medical research involving human subjects.

***Availability of data and material.*** Data-sharing agreements were jointly developed and signed by all collaborating partners.

***Disclaimer.*** The funding agency had no role in study design, data collection and analysis, decision to publish, or preparation of the manuscript.

***Supplement sponsorship.*** This supplement is sponsored by Bill & Melinda Gates Foundation.

***Financial support.*** This work was supported by a grant (INV-009018) from the Bill & Melinda Gates Foundation to the London School of Hygiene & Tropical Medicine (Principal Investigator, Joy Lawn).

***Potential conflicts of interest.*** M. W. B. and L. S. report subawards from the London School of Hygiene and Tropical Medicine (LSHTM) to D. van de Beek/M. Bijlsma Amsterdam UMC, the Netherlands, during the conduct of the study. E. H.-P. and H. T. S. report subawards from LSHTM to the Department of Clinical Epidemiology, Aarhus University Hospital (AGR-2019-731-6962). H. T. S. reports the Department of Clinical Epidemiology, Aarhus University Hospital, receives funding for other studies from companies in the form of research grants to (and administered by) Aarhus University. None of these studies have any relation to the present study. All other authors report no conflicts of interest. All authors have submitted the ICMJE Form for Disclosure of Potential Conflicts of Interest. Conflicts that the editors consider relevant to the content of the manuscript have been disclosed.

***GBS Danish and Dutch Collaborative Group for Long-term Outcomes.*** Henrik T. Sørensen (Department of Clinical Epidemiology, Aarhus University, Aarhus N, Denmark), Erzsébet Horváth-Puhó (Department of Clinical Epidemiology, Aarhus University, Aarhus N, Denmark), Kirstine K. Søgaard (Department of Clinical Epidemiology, Aarhus University, Aarhus N, Denmark; Department of Clinical Microbiology, Aalborg University Hospital, Aalborg, Denmark), Diederik van de Beek (Department of Neurology, Amsterdam Neuroscience, Amsterdam UMC, University of Amsterdam, Amsterdam, Netherlands), Merijn W. Bijlsma (Department of Neurology, Amsterdam Neuroscience, Amsterdam UMC, University of Amsterdam, Amsterdam, Netherlands), Merel N. van Kassel (Department of Neurology, Amsterdam Neuroscience, Amsterdam UMC, University of Amsterdam, Amsterdam, Netherlands), Linde Snoek (Department of Neurology, Amsterdam Neuroscience, Amsterdam UMC, University of Amsterdam, Amsterdam, Netherlands), Brechje de Gier (Centre for Infectious Disease Control, National Institute for Public Health and the Environment, Bilthoven, Netherlands), Arie van der Ende (Department of Medical Microbiology and Infection Prevention and the Netherlands Reference Laboratory for Bacterial Meningitis, Amsterdam UMC, University of Amsterdam, Amsterdam, Netherlands), and Susan J. M. Hahné (Centre for Infectious Disease Control, National Institute for Public Health and the Environment, Bilthoven, Netherlands).

## References

[1] Blencowe H , Cousens S, Oestergaard MZ, et al. National, regional, and worldwide estimates of preterm birth rates in the year 2010 with time trends since 1990 for selected countries: a systematic analysis and implications. Lancet 2012; 379:2162–72. 22682464 10.1016/S0140-6736(12)60820-4

[2] Blencowe H , Cousens S, Chou D, et al; Born Too Soon Preterm Birth Action Group. Born too soon: the global epidemiology of 15 million preterm births. Reprod Health 2013; 10(Suppl 1):S2. 24625129 10.1186/1742-4755-10-S1-S2 PMC3828585

[3] Dua T , Tomlinson M, Tablante E, et al. Global research priorities to accelerate early child development in the sustainable development era. Lancet Glob Health 2016; 4:e887–9. 27717631 10.1016/S2214-109X(16)30218-2 PMC5659186

[4] Liu L , Oza S, Hogan D, et al. Global, regional, and national causes of under-5 mortality in 2000-15: an updated systematic analysis with implications for the Sustainable Development Goals. Lancet 2016; 388:3027–35. 27839855 10.1016/S0140-6736(16)31593-8 PMC5161777

[5] Hug L , Alexander M, You D, Alkema L; UN Inter-agency Group for Child Mortality Estimation. National, regional, and global levels and trends in neonatal mortality between 1990 and 2017, with scenario-based projections to 2030: a systematic analysis. Lancet Glob Health 2019; 7:e710–e20. 31097275 10.1016/S2214-109X(19)30163-9 PMC6527519

[6] Arpino C , Compagnone E, Montanaro ML, et al. Preterm birth and neurodevelopmental outcome: a review. Childs Nerv Syst 2010; 26: 1139–49. 20349187 10.1007/s00381-010-1125-y

[7] Hee Chung E , Chou J, Brown KA. Neurodevelopmental outcomes of preterm infants: a recent literature review. Transl Pediatr 2020; 9:3–8. 10.21037/tp.2019.09.10 PMC7082240 32206579

[8] Bianchi-Jassir F , Seale AC, Kohli-Lynch M, et al. Preterm birth associated with group B Streptococcus maternal colonization worldwide: systematic review and meta-analyses. Clin Infect Dis 2017; 65:133–42. 29117329 10.1093/cid/cix661 PMC5850429

[9] Lin FY , Weisman LE, Troendle J, Adams K. Prematurity is the major risk factor for late-onset group B streptococcus disease. J Infect Dis 2003; 188:267–71. 12854082 10.1086/376457

[10] Oddie S , Embleton ND. Risk factors for early onset neonatal group B streptococcal sepsis: case-control study. BMJ 2002; 325:308. 12169506 10.1136/bmj.325.7359.308 PMC117770

[11] Madrid L , Seale AC, Kohli-Lynch M, et al; Infant GBS Disease Investigator Group. Infant group B streptococcal disease incidence and serotypes worldwide: systematic review and meta-analyses. Clin Infect Dis 2017; 65:160–72. 10.1093/cid/cix656 PMC5850457 29117326

[12] Seale AC , Bianchi-Jassir F, Russell NJ, et al. Estimates of the burden of group B streptococcal disease worldwide for pregnant women, stillbirths, and children. Clin Infect Dis 2017; 65:200–19. 10.1093/cid/cix664 PMC5849940 29117332

[13] Kohli-Lynch M , Russell NJ, Seale AC, et al. Neurodevelopmental impairment in children after group B streptococcal disease worldwide: systematic review and meta-analyses. Clin Infect Dis 2017; 65:190–9. 10.1093/cid/cix663 PMC5848372 29117331

[14] Horváth-Puhó E , van Kassel MN, Gonçalves BP, et al. Invasive group B streptococcal disease in early infancy in Denmark and the Netherlands: national cohort study of mortality, neurodevelopmental impairments, and economic outcomes. Lancet Child Adolesc Health 2021; 5:398–407. 10.1016/S2352-4642(21)00022-5 PMC8131199 33894156

[15] Kassel MNv, Gonçalves BP, Snoek LL, et al. Sex differences in long-term outcomes after Group B streptococcal infections during infancy in Denmark and the Netherlands: national cohort studies of neurodevelopmental impairments and mortality. Clin Infect Dis 2022; 74:S54–S63. 10.1093/cid/ciab822 PMC8775649 34725694

[16] Schmidt M , Schmidt SAJ, Adelborg K, et al. The Danish health care system and epidemiological research: from health care contacts to database records. Clin Epidemiol 2019; 11:563–91. 31372058 10.2147/CLEP.S179083 PMC6634267

[17] Laugesen K , Ludvigsson JF, Schmidt M, et al. Nordic Health Registry-based research: a review of health care systems and key registries. Clin Epidemiol 2021; 13:533–54. 34321928 10.2147/CLEP.S314959 PMC8302231

[18] Bliddal M , Broe A, Pottegård A, Olsen J, Langhoff-Roos J. The Danish Medical Birth Register. Eur J Epidemiol 2018; 33:27–36. 29349587 10.1007/s10654-018-0356-1

[19] Schmidt M , Pedersen L, Sørensen HT. The Danish Civil Registration System as a tool in epidemiology. Eur J Epidemiol 2014; 29:541–9. 24965263 10.1007/s10654-014-9930-3

[20] Schmidt M , Schmidt SA, Sandegaard JL, Ehrenstein V, Pedersen L, Sørensen HT. The Danish National Patient Registry: a review of content, data quality, and research potential. Clin Epidemiol 2015; 7:449–90. 26604824 10.2147/CLEP.S91125 PMC4655913

[21] Netherlands Reference Laboratory for Bacterial Meningitis (AMC/RIVM). Bacterial meningitis in The Netherlands: annual report 2016. Amsterdam, Netherlands: University of Amsterdam, 2017.

[22] Bijlsma MW , Bekker V, Brouwer MC, Spanjaard L, van de Beek D, van der Ende A. Epidemiology of invasive meningococcal disease in the Netherlands, 1960-2012: an analysis of national surveillance data. Lancet Infect Dis 2014; 14:805–12. 25104306 10.1016/S1473-3099(14)70806-0

[23] Statistics Netherlands. StatLine, The Hague/Heerlen, 2017. Available at: http://www.cbs.nl. Accessed 24 October 2017.

[24] PeriNed. Perinatale Zorg in Nederland (2000–2017) annual reports (2000–2017). 2019. Available at: https://www.perined.nl/jaarboek-zorg/jaarboekzorg-2002-2014. Accessed 14 July 2020.

[25] Knol MJ , VanderWeele TJ. Recommendations for presenting analyses of effect modification and interaction. Int J Epidemiol 2012; 41:514–20. 22253321 10.1093/ije/dyr218 PMC3324457

[26] VanderWeele TJ , Knol MJ. A tutorial on interaction. Epidemiol Methods 2014; 3:33–72.

[27] Lawn JE , Kinney M. Preterm birth: now the leading cause of child death worldwide. Sci Transl Med 2014; 6:263ed21. 10.1126/scitranslmed.aaa2563 25411468

[28] Steiner L , Diesner SC, Voitl P. Risk of infection in the first year of life in preterm children: an Austrian observational study. PLoS One 2019; 14:e0224766. 31816626 10.1371/journal.pone.0224766 PMC6901347

[29] Saini SS , Kumar P, Kumar RM. Hemodynamic changes in preterm neonates with septic shock: a prospective observational study. Pediatr Crit Care Med 2014; 15:443–50. 24717905 10.1097/PCC.0000000000000115

[30] du Plessis AJ . The role of systemic hemodynamic disturbances in prematurity-related brain injury. J Child Neurol 2009; 24:1127–40. 19745087 10.1177/0883073809339361 PMC3674829

[31] Luce WA , Hoffman TM, Bauer JA. Bench-to-bedside review: developmental influences on the mechanisms, treatment and outcomes of cardiovascular dysfunction in neonatal versus adult sepsis. Crit Care 2007; 11:228. 17903309 10.1186/cc6091 PMC2556733

[32] Babnik J , Stucin-Gantar I, Kornhauser-Cerar L, Sinkovec J, Wraber B, Derganc M. Intrauterine inflammation and the onset of peri-intraventricular hemorrhage in premature infants. Biol Neonate 2006; 90:113–21. 16549908 10.1159/000092070

[33] Al-Mouqdad MM , Abdelrahim A, Abdalgader AT, et al. Risk factors for intraventricular hemorrhage in premature infants in the central region of Saudi Arabia. Int J Pediatr Adolesc Med 2021; 8:76–81. 34084876 10.1016/j.ijpam.2019.11.005 PMC8144857

[34] Khanafer-Larocque I , Soraisham A, Stritzke A, et al. Intraventricular hemorrhage: risk factors and association with patent ductus arteriosus treatment in extremely preterm neonates. Front Pediatr 2019; 7:408. 31696098 10.3389/fped.2019.00408 PMC6817605

[35] Christian EA , Jin DL, Attenello F, et al. Trends in hospitalization of preterm infants with intraventricular hemorrhage and hydrocephalus in the United States, 2000-2010. J Neurosurg Pediatr 2016; 17:260–9. 26544084 10.3171/2015.7.PEDS15140

[36] Lawn JE , Cousens S, Zupan J; Lancet Neonatal Survival Steering Team. 4 Million neonatal deaths: when? Where? Why? Lancet 2005; 365:891–900. 15752534 10.1016/S0140-6736(05)71048-5

[37] Trijbels-Smeulders M , de Jonge GA, Pasker-de Jong PC, et al. Epidemiology of neonatal group B streptococcal disease in the Netherlands before and after introduction of guidelines for prevention. Arch Dis Child Fetal Neonatal Ed 2007; 92:F271–6. 17227807 10.1136/adc.2005.088799 PMC2675425

[38] Mukerji A , Shah V, Shah PS. Periventricular/intraventricular hemorrhage and neurodevelopmental outcomes: a meta-analysis. Pediatrics 2015; 136:1132–43. 26598455 10.1542/peds.2015-0944

[39] Blencowe H , Lee AC, Cousens S, et al. Preterm birth-associated neurodevelopmental impairment estimates at regional and global levels for 2010. Pediatr Res 2013; 74(Suppl 1):17–34. 24366461 10.1038/pr.2013.204 PMC3873710

[40] Dong Y , Chen SJ, Yu JL. A systematic review and meta-analysis of long-term development of early term infants. Neonatology 2012; 102:212–21. 22814228 10.1159/000338099

[41] Twilhaar ES , de Kieviet JF, Aarnoudse-Moens CS, van Elburg RM, Oosterlaan J. Academic performance of children born preterm: a meta-analysis and meta-regression. Arch Dis Child Fetal Neonatal Ed 2018; 103:F322–30. 28847871 10.1136/archdischild-2017-312916 PMC6047144

[42] Bekker V , Bijlsma MW, van de Beek D, Kuijpers TW, van der Ende A. Incidence of invasive group B streptococcal disease and pathogen genotype distribution in newborn babies in the Netherlands over 25 years: a nationwide surveillance study. Lancet Infect Dis 2014; 14:1083–9. 25444407 10.1016/S1473-3099(14)70919-3

[43] Ohlsson A , Shah VS. Intrapartum antibiotics for known maternal Group B streptococcal colonization. Cochrane Database Syst Rev 2013; 1:CD007467. 10.1002/14651858.CD007467.pub3 23440815

[44] Farr A , Sustr V, Kiss H, et al. Oral probiotics to reduce vaginal group B streptococcal colonization in late pregnancy. Sci Rep 2020; 10:19745. 33184437 10.1038/s41598-020-76896-4 PMC7665007

[45] Ho M , Chang YY, Chang WC, et al. Oral Lactobacillus rhamnosus GR-1 and Lactobacillus reuteri RC-14 to reduce group B Streptococcus colonization in pregnant women: a randomized controlled trial. Taiwan J Obstet Gynecol 2016; 55:515–8. 27590374 10.1016/j.tjog.2016.06.003

[46] Furfaro LL , Payne MS, Chang BJ. Host range, morphological and genomic characterisation of bacteriophages with activity against clinical Streptococcus agalactiae isolates. PLoS One 2020; 15:e0235002. 32574197 10.1371/journal.pone.0235002 PMC7310703

[47] Berardi A , Cassetti T, Creti R, et al; Prepare Network. The Italian arm of the PREPARE study: an international project to evaluate and license a maternal vaccine against group B streptococcus. Ital J Pediatr 2020; 46:160. 33115542 10.1186/s13052-020-00923-3 PMC7594470

[48] Hillier SL , Ferrieri P, Edwards MS, et al. A phase 2, randomized, control trial of group B Streptococcus (GBS) type III capsular polysaccharide-tetanus toxoid (GBS III-TT) vaccine to prevent vaginal colonization with GBS III. Clin Infect Dis 2019; 68:2079–86. 30281066 10.1093/cid/ciy838 PMC7317289

[49] van den Berg JP , Westerbeek EA, van der Klis FR, Berbers GA, van Elburg RM. Transplacental transport of IgG antibodies to preterm infants: a review of the literature. Early Hum Dev 2011; 87:67–72. 21123010 10.1016/j.earlhumdev.2010.11.003

