# Supplementary material for: Prematurity Modifies the Risk of Long-term Neurodevelopmental Impairments After Invasive Group B Streptococcus Infections During Infancy in Denmark and the Netherlands
Source: Clin Infect Dis. 2021 Nov 3;74(Suppl 1):S44–53. doi: 10.1093/cid/ciab774 (PMC8775650; doi:10.1093/cid/ciab774)
Supplement: ciab774_suppl_Supplementary_Materials [file ciab774_suppl_supplementary_materials.docx]

**SUPPLEMENTARY MATERIAL**

**Supplement Title:** Every Country, Every Woman, Every Child; Group B Streptococcal Disease Worldwide

**Paper Title:** Prematurity modifies the risk of long-term neurodevelopmental impairments after invasive Group B Streptococcus infections during infancy in Denmark and the Netherlands

**Short title:** Prematurity and outcomes of GBS infections

**Authors:** Erzsébet Horváth-Puhó PhD*^1^, Linde Snoek MD*^2^, Merel N. van Kassel MD^2^, Bronner P. Gonçalves MD^3,4^, Jaya Chandna MPhil^3,4^, Simon R. Procter DPhil^3,4^, Diederik van de Beek PhD^2^, Brechje de Gier PhD^5^, Arie van der Ende PhD^6^, Henrik Toft Sørensen MD^#1^, Joy E Lawn PhD^#3,4^, Merijn W. Bijlsma PhD^#2,7^

**Affiliations**:

^1^ Department of Clinical Epidemiology, Aarhus University, Olof Palmes Allé 43-45, 8200, Aarhus N, Denmark

^2^ Amsterdam UMC, University of Amsterdam, Department of Neurology, Amsterdam Neuroscience, Meibergdreef 9, 1100 DD Amsterdam, The Netherlands

^3^ Maternal, Adolescent, Reproductive & Child Health (MARCH) Centre, London School of Hygiene & Tropical Medicine, Keppel Street, London, WC1E 7HT, UK

^4^ Department of Infectious Disease Epidemiology, London School of Hygiene & Tropical Medicine, Keppel Street, London, WC1E 7HT, UK.

^5^ Centre for Infectious Disease Control, National Institute for Public Health and the Environment, Antonie van Leeuwenhoeklaan 9, 3721 MA Bilthoven, The Netherlands

^6^ Netherlands Reference Laboratory for Bacterial Meningitis, Amsterdam UMC/RIVM, University of Amsterdam, Department of Medical Microbiology and Infection Prevention, Amsterdam Infection and Immunity, Meibergdreef 9, 1100 DD Amsterdam, The Netherlands

^7^ Amsterdam UMC, University of Amsterdam, Department of Pediatrics, Meibergdreef 9, 1100 DD Amsterdam, The Netherlands

*equal contributions; ^#^equal contributions

Table of Contents

[***Supplementary Table 1. International Classification of Diseases codes used in Denmark to identify invasive GBS disease 3***](#_Toc81513076)

[***Supplementary Table 2. Methods used to measure effect modification 4***](#_Toc81513077)

[***Supplementary Table 3A. Effect modification of prematurity on the association between GBS sepsis and mortality 6***](#_Toc81513078)

[***Supplementary Table 4A. Effect modification by gestational age of NDI outcome after GBS sepsis. 8***](#_Toc81513079)

[***Supplementary Table 4B. Effect modification by gestational age on NDI outcome after GBS meningitis. 10***](#_Toc81513080)

[***Supplementary Table 5. Effect modification by gestational age on domain-specific NDI outcomes after invasive GBS disease in Denmark. 12***](#_Toc81513081)

[***Supplementary Figure 1. NDI outcomes in Denmark and the Netherlands among 5 and 10 year survivors: relative excess risk due to interaction. 14***](#_Toc81513082)

### Supplementary Table 1. *International Classification of Diseases* (ICD) codes used in Denmark to identify invasive GBS disease

| **Invasive GBS disease** | **ICD-10 codes** |
| --- | --- |
| **GBS sepsis** | - P36.0 (Sepsis of newborn due to GBS) - A40.1 (Sepsis due to GBS) |
| **GBS meningitis** | - G00.2 (Streptococcal meningitis) - Patients with:   {[P36.0 (Sepsis of newborn due to GBS) or  A40.1 (Sepsis due to GBS)]  **AND**  [G00.9 (Bacterial meningitis, unspecified) or  G03.9 (Meningitis, unspecified)]} |
| **GBS pneumonia** | - P23.3 (Congenital pneumonia due to GBS) - J15.3 (Pneumonia due to GBS) |

### Supplementary Table 2. Methods used to measure effect modification

| **Effect modification** | **Effect modification** occurs in biomedical research when a measure of association between an exposure and a health outcome differs according to the levels of a third variable - the effect modifier. The heterogeneity of a measure of association across the levels of an effect modifier helps identifying subgroups for which a risk factor is especially prominent, and specifying a multifactorial causal relationship. Effect modification can be measured on additive and multiplicative scale and literature suggests analysing the presence of the effect modification on both scales (VanderWeele et al, Epidemiol. Methods 2014; Knol et al, International Journal of Epidemiology 2012). There may be a positive modifying effect on the additive scale but a negative or null effect on a multiplicative scale.  The **aim of our study** was to examine whether the effect of iGBS on mortality and long-term NDI differs for preterm and term infants, and whether co-occurrence of iGBS and prematurity leads to worse outcome. As recommended by Knol et al (Knol et al, International Journal of Epidemiology 2012), we performed the following analyses:   1. Stratified analyses, where the risk of outcome variables after iGBS were analysed in term and preterm children separately; 2. Effect modification on multiplicative scale by including the product term gestational age*iGBS in regression models; 3. Analyses with common reference category using non-iGBS term children as reference group; 4. Effect modification on additive scale by calculating interaction contrasts and RERIs. |
| --- | --- |
|  |  |
| **Mortality analyses** | **Mortality risk, rate, and hazard ratios**: We assessed overall mortality by calculating mortality risk during the first 3 months and the first five years of life and mortality rates per 1000 person-years. Hazard ratios (HRs) and 95% confidence intervals (CIs) were estimated using Cox proportional hazards regression after adjusting for sex and birth year. In the stratified analyses by prematurity, mortality rates among children with a history of iGBS were compared to non-GBS children. In the analyses with a common reference category, we estimated HRs based on the reference group of non-iGBS term children.  **Effect modification**: We evaluated the extent to which gestational age modified the effect of iGBS on overall mortality on both additive and multiplicative scales. Effect modification by prematurity on the additive scale especially addresses public health-related questions, and effect modification on the multiplicative scale quantifies whether relative associations between iGBS and mortality depend on the child’s gestational age.  **Additive scale:** Effect modification on the additive scale was examined by calculating the ***interaction contrast***. An interaction contrast is a measure of the departure of mortality rates from an additive model. It is calculated as the difference between rate differences in strata with and without prematurity, as follows*:* ***IC = (Mortality rate_iGBS, Preterm_ – Mortality rate_non-iGBS, Preterm_) – (Mortality rate_IGBS, Term_ – Mortality rate_non-IGBS, Term_)***. In addition, we calculated ***attributable proportions*** (= interaction contrast/mortality rate in preterm children with iGBS).  **Multiplicative scale:** On the multiplicative scale, a modification occurs if relative association measures (i.e., HRs) between exposure and outcome vary by strata of a third variable. We therefore included the product term (gestational age*iGBS) in multivariable Cox regression models. |
|  |  |
| **NDI analyses** | **NDI and special education risk and odd ratios**: Risks of NDI and need for special education were assessed at the ages of 5 and 10 years. The analyses included only those children followed until at least the corresponding cutoff age. The association between iGBS and NDI was assessed using logistic regression models; estimated odds ratios (ORs) and 95% CIs were adjusted for year of birth and sex. In the stratified analyses by prematurity, NDI risk among children with a history of iGBS were compared to non-GBS children. In the analyses with a common reference category, we estimated ORs based on the reference group of non-iGBS term children.  **Effect modification**: We assessed whether gestational age modified the effect of iGBS on NDI outcomes on both additive and multiplicative scales.  **Additive scale:** Effect modification on the additive scale was examined by calculating the ***relative excess risk due to interaction*** (RERI), using the common reference group of term non-iGBS children and the ORs estimated in these analyses (***RERI = OR_iGBS, Preterm_ – OR_iGBS, Term_ – OR_non-IGBS, Preterm_ + 1***). In addition, we calculated ***attributable proportions*** as RERI / OR_iGBS, Preterm_.  **Multiplicative scale:** On the multiplicative scale, a modification occurs if ORs between iGBS and NDI vary by strata of a gestational age. We therefore included the product term (gestational age*iGBS) in the logistic regression models. |

### Supplementary Table 3A. Effect modification of prematurity on the association between GBS sepsis and mortality

|  |  | **ANALYSES WITH A COMMON REFERENCE GROUP** | | **STRATIFIED ANALYSES** | |
| --- | --- | --- | --- | --- | --- |
| **Age of children** | **Mortality rate**  **per 1000 PYs** | **HRs (95% CI) for iGBS**  **using a common reference group** | **Effect modification on additive scale**  ***(Interaction contrast [95%CI]; AP[%])*** | **HRs (95% CI) for iGBS**  **within strata of gestational age** | **Effect modification on**  **multiplicative scale**  ***(p-value of***  ***iGBS*prematurity term)*** |
| **DENMARK** |  |  |  |  |  |
| **0-89d** |  |  |  |  |  |
| iGBS-/Term | 4.5 (1.8–7.2) | *1.00 (reference)* |  | *1.00 (reference)* |  |
| iGBS+/Term | 33.0 (10.1–55.8) | 7.30 (2.94–18.14) |  | 7.30 (2.94–18.16) |  |
| iGBS-/Preterm | 369.1 (318.8–419.4) | 78.76 (42.94–144.46) |  | *1.00 (reference)* |  |
| iGBS+/Preterm | 319.5 (175.9–463.2) | 68.11 (32.41–143.13) | *-78.1 (-232.0*–*75.9); N.A.* | 0.86 (0.54–1.38) | *p < 0.0001* |
| **0-5y** |  |  |  |  |  |
| iGBS-/Term | 0.7 (0.4–0.9) | *1.00 (reference)* |  | *1.00 (reference)* |  |
| iGBS+/Term | 1.92 (0.7–3.9) | 2.92 (1.39–6.12) |  | 2.92 (1.39–6.14) |  |
| iGBS-/Preterm | 20.3 (17.6–23.0) | 29.88 (20.51–43.54) |  | *1.00 (reference)* |  |
| iGBS+/Preterm | 17.56 (9.9–25.3) | 25.61 (14.60–44.93) | *-4.0 (-12.2*–*4.3); N.A.* | 0.86 (0.54–1.35) | *p = 0.006* |
| **THE NETHERLANDS** |  |  |  |  |  |
| **0–89d** |  |  |  |  |  |
| iGBS-/Term | 5.6 (0.7–10.6) | *1.00 (reference)* |  | *1.00 (reference)* |  |
| iGBS+/Term | 138.9 (60.3–217.5) | 24.20 (8.52–68.68) |  | 24.33 (8.57–69.05) |  |
| iGBS-/Preterm | 300.1 (238.7–361.4) | 51.60 (20.97–126.97) |  | *1.00 (reference)* |  |
| iGBS+/Preterm | 701.3 (393.9–1008.6) | 114.11 (42.81–304.12) | *267.9 (-55.2–591.1); 38%* | 2.21 (1.36–3.58) | *p < 0.0001* |
| **0–5y** |  |  |  |  |  |
| iGBS-/Term | 0.5 (0.2–0.9) | *1.00 (reference)* |  | *1.00 (reference)* |  |
| iGBS+/Term | 8.8 (4.0–13.6) | 15.66 (6.49–37.78) |  | 15.66 (6.49–37.79) |  |
| iGBS-/Preterm | 18.9 (15.2–22.7) | 32.01 (15.56–65.88) |  | *1.00 (reference)* |  |
| iGBS+/Preterm | 45.3 (25.9–64.6) | 68.36 (30.25–154.48) | *18.1 (-2.3*–*38.4); 40%* | 2.13 (1.33–3.41) | *p < 0.0001* |

Abbreviations: PY = person-year; HR = hazard ratio; iGBS = invasive GBS disease; CI = confidence interval; AP = attributable proportion

Hazard ratios were adjusted for birth year and sex.

**Supplementary Table 3B. Effect modification of prematurity on the association between GBS meningitis and mortality (the Netherlands only).**

|  |  | **ANALYSES WITH A COMMON REFERENCE GROUP** | | **STRATIFIED ANALYSES** | |
| --- | --- | --- | --- | --- | --- |
| **Age of children** | **Mortality rate**  **per 1000 PYs** | **HRs (95% CI) for iGBS**  **using a common reference group** | **Effect modification on additive scale**  ***(Interaction contrast [95%CI]; AP[%])*** | **HRs (95% CI) for iGBS**  **within strata of gestational age** | **Effect modification on**  **multiplicative scale**  ***(p-value of***  ***iGBS*prematurity term)*** |
| **THE NETHERLANDS** |  |  |  |  |  |
| **0–89d** |  |  |  |  |  |
| iGBS-/Term | 10.8 (0.2–21.4) | *1.00 (reference)* |  | *1.00 (reference)* |  |
| iGBS+/Term | 376.3 (171.7–580.8) | 33.32 (10.87–102.21) |  | 33.59 (10.95–103.02) |  |
| iGBS-/Preterm | 177.5 (97.7–257.3) | 16.02 (5.45–47.11) |  | *1.00 (reference)* |  |
| iGBS+/Preterm | 587.7 (117.4–1057.9) | 50.78 (14.33–179.98) | *44.76 (-474.31*–*563.82); 8%* | 3.16 (1.26–7.92) | *p = 0.002* |
| **0–5y** |  |  |  |  |  |
| iGBS-/Term | 0.9 (0.2–1.6) | *1.00 (reference)* |  | *1.00 (reference)* |  |
| iGBS+/Term | 21.4 (9.7–33.0) | 21.74 (8.26–57.19) |  | 21.74 (8.26–57.22) |  |
| iGBS-/Preterm | 11.4 (6.7–16.2) | 12.67 (5.14–31.25) |  | *1.00 (reference)* |  |
| iGBS+/Preterm | 33.3 (6.7–60.0) | 31.19 (10.05–96.79) | *1.46 (-28.02*–*30.95); 4%* | 2.46 (1.00–6.08) | *p = 0.001* |

Abbreviations: PY = person-year; HR = hazard ratio; iGBS = invasive GBS disease; CI = confidence interval; AP = attributable proportion

Hazard ratios were adjusted for birth year and sex.

### Supplementary Table 4A. Effect modification by gestational age of NDI outcome after GBS sepsis.

|  |  | **ANALYSES WITH A COMMON REFERENCE GROUP** | | **STRATIFIED ANALYSES** | |
| --- | --- | --- | --- | --- | --- |
| **Age of children** | **NDI proportion (%, 95% CI)** | **ORs (95% CI) for iGBS**  **using a common**  **reference group** | **Effect modification on**  **additive scale**  ***(RERI [95%CI]; AP[%])*** | **ORs (95% CI) for iGBS within**  **strata of gestational age** | **Effect modification on multiplicative**  **scale**  ***(p-value of***  ***iGBS*prematurity term)*** |
| **DENMARK** |  |  |  |  |  |
| **5 years** |  |  |  |  |  |
| **Any** |  |  |  |  |  |
| iGBS-/Term | 1.5 (1.2-1.7) | 1.00 (*reference*) |  | 1.00 (*reference*) |  |
| iGBS+/Term | 2.6 (1.6-3.9) | 1.80 (1.14–2.85) |  | 1.80 (1.14–2.85) |  |
| iGBS-/Preterm | 3.7 (2.9-4.7) | 2.61 (1.94–3.52) |  | 1.00 (*reference*) |  |
| iGBS+/Preterm | 7.1 (4.0-11.5) | 5.26 (3.02–9.18) | *1.85 (-1.10*–*4.80); 35%* | 2.02 (1.13–3.60) | *p = 0.77* |
| **Mod-sev** |  |  |  |  |  |
| iGBS-/Term | 0.8 (0.6-1.0) | 1.00 (*reference*) |  | 1.00 (*reference*) |  |
| iGBS+/Term | 1.6 (0.9-2.8) | 2.13 (1.19–3.81) |  | 2.14 (1.19–3.82) |  |
| iGBS-/Preterm | 2.2 (1.6-3.0) | 2.87 (1.94–4.24) |  | 1.00 (*reference*) |  |
| iGBS+/Preterm | 4.3 (2.0-7.9) | 5.70 (2.80–11.63) | *1.70 (-2.39*–*5.79); 30%* | 1.97 (0.94–4.12) | *p = 0.88* |
| **10 years** |  |  |  |  |  |
| **Any** |  |  |  |  |  |
| iGBS-/Term | 4.8 (4.3-5.4) | 1.00 (*reference*) |  | 1.00 (*reference*) |  |
| iGBS+/Term | 7.8 (5.8-10.2) | 1.69 (1.23–2.32) |  | 1.69 (1.23–2.32) |  |
| iGBS-/Preterm | 7.6 (6.3-9.1) | 1.66 (1.32–2.09) |  | 1.00 (*reference*) |  |
| iGBS+/Preterm | 9.5 (5.4-15.2) | 2.12 (1.23–3.68) | *-0.23 (-1.52*–*1.07); N.A.* | 1.28 (0.72–2.26) | *p = 0.40* |
| **Mod-sev** |  |  |  |  |  |
| iGBS-/Term | 2.0 (1.7-2.4) | 1.00 (*reference*) |  | 1.00 (*reference*) |  |
| iGBS+/Term | 3.3 (2.1-5.1) | 1.72 (1.07–2.76) |  | 1.72 (1.07–2.76) |  |
| iGBS-/Preterm | 3.7 (2.8-4.9) | 1.91 (1.38–2.65) |  | 1.00 (*reference*) |  |
| iGBS+/Preterm | 4.4 (1.8-8.9) | 2.29 (1.05–5.01) | *-0.34 (-2.33*–*1.64); N.A.* | 1.19 (0.53–2.68) | *p = 0.45* |
| **THE NETHERLANDS** |  |  |  |  |  |
| **5 yr** |  |  |  |  |  |
| **Any** |  |  |  |  |  |
| iGBS-/Term | 0.7 (0.4-0.1) | 1.00 (*reference*) |  | 1.00 (*reference*) |  |
| iGBS+/Term | 2.3 (0.9-5.0) | 3.39 (1.33–8.63) |  | 3.38 (1.33–8.62) |  |
| iGBS-/Preterm | 2.2 (1.3-3.5) | 3.38 (1.70–6.72) |  | 1.00 (*reference*) |  |
| iGBS+/Preterm | 3.8 (0.8-10.7) | 5.91 (1.68–20.70) | *0.14 (*-*7.44*–*7.73); 2%* | 1.73 (0.49–6.16) | *p = 0.41* |
| **Mod–sev** |  |  |  |  |  |
| iGBS-/Term | 0.4 (0.2-0.7) | 1.00 (*reference*) |  | 1.00 (*reference*) |  |
| iGBS+/Term | 2.0 (0.6-4.5) | 5.65 (1.87–17.02) |  | 5.63 (1.87–16.96) |  |
| iGBS-/Preterm | 1.8 (0.9-3.0) | 5.09 (2.14–12.08) |  | 1.00 (*reference*) |  |
| iGBS+/Preterm | 3.8 (0.8-10.7) | 10.97 (2.88–41.83) | *1.23 (*-*12.75*–*15.22); 11%* | 2.15 (0.59–7.84) | *p = 0.94* |
| **10 years** |  |  |  |  |  |
| **Any** |  |  |  |  |  |
| iGBS-/Term | 5.1 (4.0-6.5) | 1.00 (*reference*) |  | 1.00 (*reference*) |  |
| iGBS+/Term | 10.7 (6.0-17.3) | 2.27 (1.23–4.20) |  | 2.29 (1.23–4.24) |  |
| iGBS-/Preterm | 10.0 (7.1-13.6) | 2.29 (1.49–3.53) |  | 1.00 (*reference*) |  |
| iGBS+/Preterm | 18.4 (7.7-34.3) | 4.97 (2.05–12.06) | *1.41 (*-*3.08*–*5.89); 28%* | 2.11 (0.85–5.21) | *p = 0.26* |
| **Mod–sev** |  |  |  |  |  |
| iGBS-/Term | 2.5 (1.7-3.5) | 1.00 (*reference*) |  | 1.00 (*reference*) |  |
| iGBS+/Term | 6.9 (3.2-12.6) | 2.92 (1.35–6.31) |  | 2.94 (1.36–6.38) |  |
| iGBS-/Preterm | 5.3 (3.2-8.1) | 2.43 (1.36–4.37) |  | 1.00 (*reference*) |  |
| iGBS+/Preterm | 10.5 (2.9-24.8) | 5.46 (1.78–16.79) | *1.11 (*-*5.16*–*7.37); 20%* | 2.19 (0.69–6.91) | *p = 0.71* |

Abbreviations: NDI = neurodevelopmental impairment; OR = hazard ratio; iGBS = invasive GBS disease; RERI = relative risk due to interaction; CI = confidence interval; AP = attributable proportion

Odds ratios were adjusted for birth year and sex.

### Supplementary Table 4B. Effect modification by gestational age on NDI outcome after GBS meningitis.

|  |  | **ANALYSES WITH A COMMON REFERENCE GROUP** | | **STRATIFIED ANALYSES** | |
| --- | --- | --- | --- | --- | --- |
| **Age of children** | **NDI proportion (95% CI)** | **ORs (95% CI) for iGBS**  **using a common**  **reference group** | **Effect modification on**  **additive scale**  ***(RERI [95%CI]; AP[%])*** | **ORs (95% CI) for iGBS within**  **strata of gestational age** | **Effect modification on multiplicative**  **scale**  ***(p-value of***  ***iGBS*prematurity term)*** |
| **DENMARK** |  |  |  |  |  |
| **5 years** |  |  |  |  |  |
| **Any** |  |  |  |  |  |
| iGBS-/Term | 0.9 (0.4-1.8) | 1.00 (*reference*) |  | 1.00 (*reference*) |  |
| iGBS+/Term | 14.5 (7.7-23.9) | 18.16 (7.18–45.92) |  | 18.05 (7.14–45.64) |  |
| iGBS-/Preterm | 4.2 (2.4-6.8) | 4.80 (2.02–11.41) |  | 1.00 (*reference*) |  |
| iGBS+/Preterm | 17.9 (7.5-33.5) | 23.93 (8.12–70.53) | *1.97 (-20.78*–*24.72); 8%* | 5.00 (1.91–13.09) | *p = 0.06* |
| **Mod-sev** |  |  |  |  |  |
| iGBS-/Term | 0.5 (0.1-1.2) | 1.00 (*reference*) |  | 1.00 (*reference*) |  |
| iGBS+/Term | 10.8 (5.1-19.6) | 26.65 (7.99–88.86) |  | 26.68 (7.98–89.14) |  |
| iGBS-/Preterm | 3.2 (1.6-5.5) | 7.70 (2.44–24.33) |  | 1.00 (*reference*) |  |
| iGBS+/Preterm | 12.8 (4.3-27.4) | 34.76 (8.81–137.12) | *1.41 (-36.63*–*39.48); 4%* | 4.52 (1.50–13.63) | *p = 0.03* |
| **10 years** |  |  |  |  |  |
| **Any** |  |  |  |  |  |
| iGBS-/Term | 4.3 (2.8-6.3) | 1.00 (*reference*) |  | 1.00 (*reference*) |  |
| iGBS+/Term | 21.1 (11.4-33.9) | 5.97 (2.80–12.72) |  | 5.95 (2.80–12.67) |  |
| iGBS-/Preterm | 11.2 (7.7-15.5) | 2.64 (1.52–4.58) |  | 1.00 (*reference*) |  |
| iGBS+/Preterm | 27.6 (12.7-47.2) | 8.04 (3.23–20.02) | *0.42 (-7.26*–*8.10); 5%* | 3.05 (1.24–7.52) | *p = 0.26* |
| **Mod-sev** |  |  |  |  |  |
| iGBS-/Term | 2.4 (1.3-4.0) | 1.00 (*reference*) |  | 1.00 (*reference*) |  |
| iGBS+/Term | 12.3 (5.1-23.7) | 5.67 (2.19–14.70) |  | 5.66 (2.18–14.69) |  |
| iGBS-/Preterm | 6.5 (3.9-10.0) | 2.72 (1.33–5.59) |  | 1.00 (*reference*) |  |
| iGBS+/Preterm | 20.7 (8.0-39.7) | 10.25 (3.60–29.21) | *2.86 (-7.54*–*13.26); 28%* | 3.76 (1.36–10.42) | *p = 0.57* |
| **THE NETHERLANDS** |  |  |  |  |  |
| **5 yr** |  |  |  |  |  |
| **Any** |  |  |  |  |  |
| iGBS-/Term | 0.9 (0.4-1.7) | 1.00 (*reference*) |  | 1.00 (*reference*) |  |
| iGBS+/Term | 2.8 (0.6-8.1) | 3.06 (0.82-11.35) |  | 3.06 (0.82-11.44) |  |
| iGBS-/Preterm | 2.7 (1.2-5.3) | 2.99 (1.17-7.69) |  | 1.00 (*reference*) |  |
| iGBS+/Preterm | 21.9 (9.3-40.0) | 33.73 (11.42-99.67) | *28.69 (-6.07*–*63.45); 85%* | 10.74 (3.50-32.95) | *p = 0.14* |
| **Mod–sev** |  |  |  |  |  |
| iGBS-/Term | 0.7 (0.2-1.3) | 1.00 (*reference*) |  | 1.00 (*reference*) |  |
| iGBS+/Term | 2.8 (0.6-8.1) | 4.42 (1.12-17.44) |  | 4.45 (1.12-17.69) |  |
| iGBS-/Preterm | 2.7 (1.2-5.3) | 4.29 (1.54-11.98) |  | 1.00 (*reference*) |  |
| iGBS+/Preterm | 15.6 (5.3-32.8) | 30.32 (8.85-103.91) | *22.61 (-11.74*–*56.95); 75%* | 6.78 (2.06-22.36) | *p = 0.54* |
| **10 years** |  |  |  |  |  |
| **Any** |  |  |  |  |  |
| iGBS-/Term | 5.3 (3.8-7.3) | 1.00 (*reference*) |  | 1.00 (*reference*) |  |
| iGBS+/Term | 14.1 (6.6-25.0) | 2.91 (1.32-6.40) |  | 2.91 (1.32-6.41) |  |
| iGBS-/Preterm | 9.8 (5.9-15.1) | 2.08 (1.14-3.81) |  | 1.00 (*reference*) |  |
| iGBS+/Preterm | 31.6 (12.6-56.6) | 9.25 (3.22-26.59) | *5.27 (-4.36*–*14.89); 57%* | 4.40 (1.46-13.29) | *p = 0.62* |
| **Mod–sev** |  |  |  |  |  |
| iGBS-/Term | 3.1 (1.9-4.7) | 1.00 (*reference*) |  | 1.00 (*reference*) |  |
| iGBS+/Term | 6.3 (1.7-15.2) | 2.06 (0.68-6.24) |  | 2.06 (0.68-6.27) |  |
| iGBS-/Preterm | 6.6 (3.4-11.2) | 2.36 (1.12-4.99) |  | 1.00 (*reference*) |  |
| iGBS+/Preterm | 31.6 (12.6-56.6) | 16.93 (5.57-51.48) | *13.51 (-4.78*–*31.80); 79%* | 7.04 (2.20-22.55) | *p = 0.13* |

Abbreviations: NDI = neurodevelopmental impairment; OR = hazard ratio; iGBS = invasive GBS disease; RERI = relative risk due to interaction; CI = confidence interval; AP = attributable proportion

Odds ratios were adjusted for birth year and sex.

### Supplementary Table 5. Effect modification by gestational age on domain-specific NDI outcomes after invasive GBS disease in Denmark.

|  |  | **ANALYSES WITH A COMMON REFERENCE GROUP** | | **STRATIFIED ANALYSES** | |
| --- | --- | --- | --- | --- | --- |
| **Age of children** | **NDI proportion (95% CI)** | **ORs (95% CI) for iGBS**  **using a common**  **reference group** | **Effect modification on**  **additive scale**  ***(RERI [95%CI]; AP[%])*** | **ORs (95% CI) for iGBS within**  **strata of gestational age** | **Effect modification on multiplicative**  **scale**  ***(p-value of***  ***iGBS*prematurity term)*** |
| **DENMARK** |  |  |  |  |  |
| **5 years** |  |  |  |  |  |
| **Visual** |  |  |  |  |  |
| iGBS-/Term | 0.2 (0.1-0.3) | 1.00 (*reference*) |  | 1.00 (*reference*) |  |
| iGBS+/Term | 0.5 (0.2-1.3) | 2.82 (1.04–7.61) |  | 2.81 (1.04–7.60) |  |
| iGBS-/Preterm | 0.6 (0.3-1.0) | 3.03 (1.48–6.19) |  | 1.00 (*reference*) |  |
| iGBS+/Preterm | 1.2 (0.3-3.5) | 6.42 (1.88–21.96) | *1.58 (-6.32*–*9.48); 25%* | 2.14 (0.60–7.56) | *p = 0.73* |
| **Hearing** |  |  |  |  |  |
| iGBS-/Term | 0.2 (0.1-0.3) | 1.00 (*reference*) |  | 1.00 (*reference*) |  |
| iGBS+/Term | 0.5 (0.2-1.3) | 2.82 (1.04–7.61) |  | 2.81 (1.04–7.60) |  |
| iGBS-/Preterm | 0.4 (0.2-0.8) | 2.05 (0.92–4.58) |  | 1.00 (*reference*) |  |
| iGBS+/Preterm | 0.8 (0.1-2.9) | 4.19 (0.97–18.20) | *0.32 (-6.17–6.82); 8%* | 2.05 (0.44–9.56) | *p = 0.73* |
| **Cognitive** |  |  |  |  |  |
| iGBS-/Term | 0.8 (0.6-1.0) | 1.00 (*reference*) |  | 1.00 (*reference*) |  |
| iGBS+/Term | 1.4 (0.7-2.4) | 1.87 (1.03–3.38) |  | 1.87 (1.03–3.39) |  |
| iGBS-/Preterm | 1.4 (0.9-1.9) | 1.83 (1.20–2.81) |  | 1.00 (*reference*) |  |
| iGBS+/Preterm | 2.8 (1.1-5.7) | 3.86 (1.76–8.50) | *1.16 (-1.99–4.32); 30%* | 2.11 (0.91–4.85) | *p = 0.82* |
| **Social** |  |  |  |  |  |
| iGBS-/Term | 0.5 (0.4-0.6) | 1.00 (*reference*) |  | 1.00 (*reference*) |  |
| iGBS+/Term | 0.9 (0.4-1.7) | 1.80 (0.85–3.84) |  | 1.80 (0.85–3.84) |  |
| iGBS-/Preterm | 1.1 (0.7-1.6) | 2.25 (1.37–3.70) |  | 1.00 (*reference*) |  |
| iGBS+/Preterm | 3.6 (1.7-6.7) | 7.93 (3.83–16.41) | *4.87 (-0.75–10.49); 61%* | 3.53 (1.62–7.68) | *p = 0.23* |
| **Motor** |  |  |  |  |  |
| iGBS-/Term | 0.2 (0.1-0.3) | 1.00 (*reference*) |  | 1.00 (*reference*) |  |
| iGBS+/Term | 1.8 (1.1-2.9) | 8.75 (4.57–16.76) |  | 8.76 (4.57–16.79) |  |
| iGBS-/Preterm | 1.8 (1.3-2.4) | 8.74 (5.11–14.96) |  | 1.00 (*reference*) |  |
| iGBS+/Preterm | 4.8 (2.5-8.2) | 24.07 (11.62–49.85) | *7.58 (-7.54–22.70); 31%* | 2.74 (1.42–5.29) | *p = 0.01* |
| **10 years** |  |  |  |  |  |
| **Visual** |  |  |  |  |  |
| iGBS-/Term | 0.4 (0.2-0.5) | 1.00 (*reference*) |  | 1.00 (*reference*) |  |
| iGBS+/Term | 0.9 (0.3-1.9) | 2.51 (1.02–6.17) |  | 2.51 (1.02–6.17) |  |
| iGBS-/Preterm | 0.7 (0.4-1.2) | 2.00 (1.00–4.02) |  | 1.00 (*reference*) |  |
| iGBS+/Preterm | 1.6 (0.3-4.6) | 4.66 (1.39–15.63) | *1.14 (-4.66–6.95); 24%* | 2.33 (0.65–8.34) | *p = 0.92* |
| **Hearing** |  |  |  |  |  |
| iGBS-/Term | 0.5 (0.3-0.6) | 1.00 (*reference*) |  | 1.00 (*reference*) |  |
| iGBS+/Term | 1.0 (0.4-2.1) | 2.27 (1.00–5.18) |  | 2.27 (1.00–5.18) |  |
| iGBS-/Preterm | 0.5 (0.2-1.0) | 1.16 (0.55–2.45) |  | 1.00 (*reference*) |  |
| iGBS+/Preterm | 1.6 (0.3-4.6) | 3.62 (1.10–11.96) | *1.19 (-3.34–5.72); 33%* | 3.11 (0.83–11.64) | *p = 0.69* |
| **Cognitive** |  |  |  |  |  |
| iGBS-/Term | 2.4 (2.1-2.8) | 1.00 (*reference*) |  | 1.00 (*reference*) |  |
| iGBS+/Term | 3.8 (2.5-5.5) | 1.60 (1.05–2.45) |  | 1.60 (1.05–2.45) |  |
| iGBS-/Preterm | 3.9 (3.0-4.9) | 1.65 (1.24–2.21) |  | 1.00 (*reference*) |  |
| iGBS+/Preterm | 3.7 (1.5-7.6) | 1.59 (0.73–3.44) | *-0.67 (-2.11–0.77); N.A.* | 0.96 (0.43–2.13) | *p = 0.26* |
| **Social** |  |  |  |  |  |
| iGBS-/Term | 3.0 (2.6-3.5) | 1.00 (*reference*) |  | 1.00 (*reference*) |  |
| iGBS+/Term | 5.3 (3.7-7.2) | 1.79 (1.24–2.58) |  | 1.79 (1.24–2.58) |  |
| iGBS-/Preterm | 4.8 (3.8-5.9) | 1.61 (1.24–2.10) |  | 1.00 (*reference*) |  |
| iGBS+/Preterm | 5.9 (3.0-10.3) | 2.02 (1.08–3.79) | *-0.38 (-1.82–1.06); N.A.* | 1.25 (0.65–2.41) | *p = 0.35* |
| **Motor** |  |  |  |  |  |
| iGBS-/Term | 0.4 (0.3-0.6) | 1.00 (*reference*) |  | 1.00 (*reference*) |  |
| iGBS+/Term | 1.8 (0.9-3.0) | 4.68 (2.35–9.32) |  | 4.70 (2.36–9.36) |  |
| iGBS-/Preterm | 2.1 (1.5-2.9) | 5.67 (3.42–9.43) |  | 1.00 (*reference*) |  |
| iGBS+/Preterm | 5.3 (2.6-9.6) | 15.05 (7.14–31.71) | *5.70 (-4.60–16.00); 38%* | 2.65 (1.29–5.42) | *p = 0.26* |

Abbreviations: NDI = neurodevelopmental impairment; OR = hazard ratio; iGBS = invasive GBS disease; RERI = relative risk due to interaction; CI = confidence interval; AP = attributable proportion

Odds ratios were adjusted for birth year and sex.


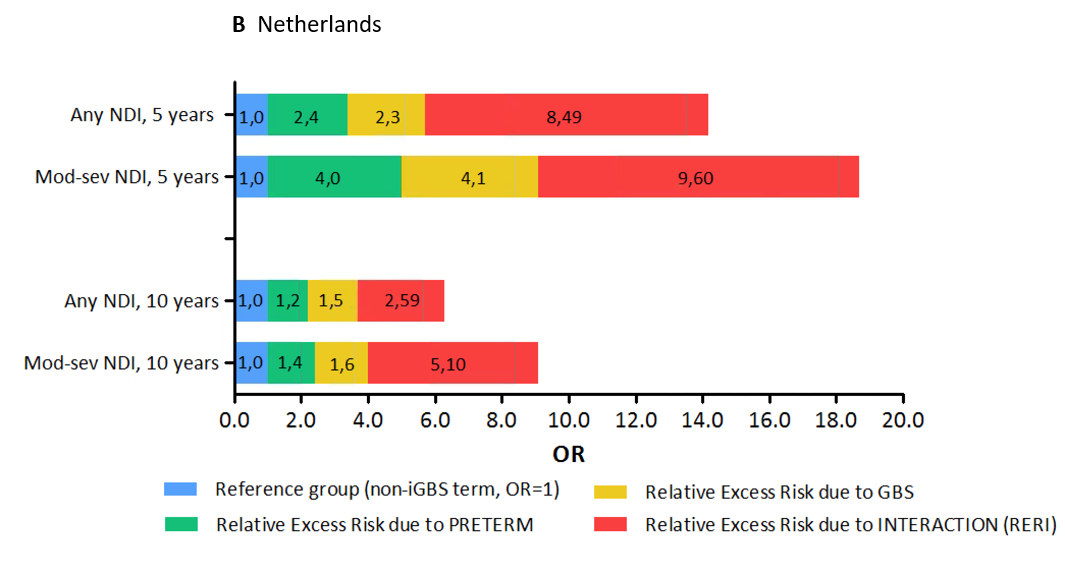

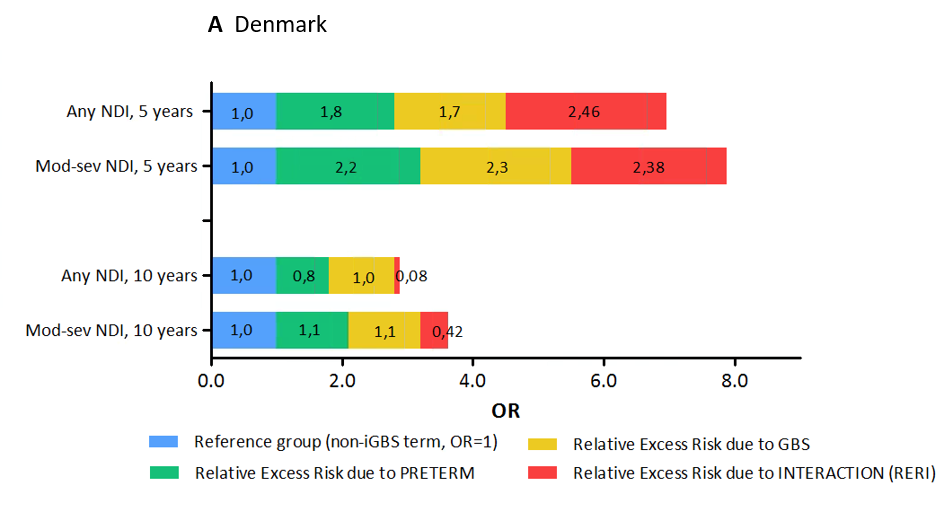
Supplementary Figure 1. NDI outcomes in Denmark and the Netherlands among 5 and 10 year survivors: relative excess risk due to interaction.

The blue bar corresponds to the non-iGBS term children (*OR = 1*). The green bar displays the excess relative risk due to prematurity (non-iGBS preterm vs. non-iGBS term; *OR_non-IGBS, Preterm_ – 1)*. The orange bar corresponds to the excess relative risk due to iGBS alone (iGBS term vs. non-iGBS term; *OR_iGBS, Term_ – 1*). The red bar displays the excess relative risk due to interaction (*RERI = OR_iGBS, Preterm_ – OR_iGBS, Term_ – OR_non-IGBS, Preterm_ + 1*). The combination of the blue, green, orange, and red bars indicate the odds ratio comparing the iGBS preterm children vs. non-iGBS term children; *OR_iGBS, Preterm_*).
